# The Development of a Specific Nanofiber Bioreceptor for Detection of *Escherichia coli* and *Staphylococcus aureus* from Air

**DOI:** 10.3390/bios14050234

**Published:** 2024-05-08

**Authors:** Leontýna Varvařovská, Petr Kudrna, Bruno Sopko, Taťána Jarošíková

**Affiliations:** 1 Department of Natural Sciences, Faculty of Biomedical Engineering, Czech Technical University in Prague, 272 01 Kladno, Czech Republic; kudrnpet@fbmi.cvut.cz (P.K.); jarostat@fbmi.cvut.cz (T.J.); 2 Laboratory of Advanced Biomaterials, University Centre for Energy Efficient Buildings, Czech Technical University in Prague, 273 43 Buštěhrad, Czech Republic; bruno.sopko@lfmotol.cuni.cz; 3 Department of Medical Chemistry and Biomedical Biochemistry, Second Faculty of Medicine, Charles University, 150 00 Prague, Czech Republic

**Keywords:** nanofibers, nanofiber biosensor, immuno-nanosensor, bacterial detection

## Abstract

Polluted air and the presence of numerous airborne pathogens affect our daily lives. The sensitive and fast detection of pollutants and pathogens is crucial for environmental monitoring and effective medical diagnostics. Compared to conventional detection methods (PCR, ELISA, metabolic tests, etc.), biosensors bring a very attractive possibility to detect chemicals and organic particles with the mentioned reliability and sensitivity in real time. Moreover, by integrating nanomaterials into the biosensor structure, it is possible to increase the sensitivity and specificity of the device significantly. However, air quality monitoring could be more problematic even with such devices. The greatest challenge with conservative and sensing methods for detecting organic matter such as bacteria is the need to use liquid samples, which slows down the detection procedure and makes it more difficult. In this work, we present the development of a polyacrylonitrile nanofiber bioreceptor functionalized with antibodies against bacterial antigens for the specific interception of bacterial cells directly from the air. We tested the presented novel nanofiber bioreceptor using a unique air filtration system we had previously created. The prepared antibody-functionalized nanofiber membranes for air filtration and pathogen detection (with model organisms *E. coli* and *S. aureus*) show a statistically significant increase in bacterial interception compared to unmodified nanofibers. Creating such a bioreceptor could lead to the development of an inexpensive, fast, sensitive, and incredibly selective bionanosensor for detecting bacterial polluted air in commercial premises or medical facilities.

## 1. Introduction

Nowadays, the rapid and eminent development of biomedicine and environmental monitoring is mainly due to the possibility of easy, fast, precise, and sensitive diagnostics and detection [1,2,3]. For such a development, sensors are the tools of great interest. In addition, combined with bioactive molecules (antibodies, enzymes, nucleic acids, etc.), (bio)sensors allow for the reliable detection of different biological and chemical markers. The main attractivity of biosensors stands especially on particular and sensitive biological interactions between analytes and the recognition bioactive element of the sensor (so-called bioreceptor) [1]. The most common biosensors commercially used are glucometers—sensors for glucose monitoring in blood [4,5]. However, in addition to monitoring and detecting glucose and other chemical analytes and biomarkers (hormones, enzymes, lipids, etc.), fast and so-called online detection of pathogens is also a significant priority.

Biosensors have become an exciting alternative to pathogen detection in microbiology and epidemiology. Today, the most common methods for determining bacteria and viruses are ELISA, PCR, and metabolic tests [6,7,8,9]. However, biosensors reduce costs (in some cases up to 96% [9], but on average, around 40% [10] and time (from hours with PCR to units to tens of minutes with biosensors [11]). Among other things, device sensitivity can be increased by incorporating nanomaterials [12,13,14,15,16,17,18] into the biosensor system, and it is possible to achieve LoD in fM concentration [12,13]. This increase in sensitivity is secured mainly using nanofibers. Their characteristic structure with the immense number of pores [19,20,21] creates an enormous active surface that can be modified, enriched, or functionalized [22,23].

Functionalization is a process of immobilizing bioactive molecules in the matrix structure [24]. Nanofibers modified by this process are the subject of recent studies. Whether it is functionalization with nucleic acids (such as DNA immobilization for the detection of Salmonella [25]) or antibodies (specific antibodies against Pseudomonas aeruginosa [26], Helicobacter pylori [27], or Streptococcus agalactiae [28]), low detection limits in the units of CFU·mL^−1^, high sensitivity, and fast response characterize these biosensors. These mentioned studies are dedicated to pathogen detection from liquid samples. However, many pathogens are transmitted through the air, and in addition to causing respiratory diseases, they also cause nosocomial infections. Pathogen detection directly from the air is becoming an attractive and desired method for the environmental monitoring of polluted air. Although many different biosensors exist, their use for detecting analytes from air faces challenges in bioreceptor preservation [29,30,31,32]. Nevertheless, pathogen monitoring in the air could help prevent respiratory disease epidemics or the emergence of nosocomial infections in operating rooms, intensive units, and hospitals in general.

The main goal of this work was to prepare antibody-functionalized nanofibers as bioreceptors for the interception and detection of selected bacterial organisms. In this work, we present the needleless electrospinning process of polyacrylonitrile nanofiber fabrication; the process of their functionalization; and finally, the evaluation of the bioreceptor’s bacterial interception effectiveness through optical density measurement. This work is directly linked to the conference paper from the EHB 2023 conference but expands the mentioned paper with more detailed methodology and new results (supplemented results of detecting *E. coli* and added new results of detecting *S. aureus*) [33].

## 2. Materials and Methods

Considering nanofibers’ characteristic structure, a mechanically and chemically durable synthetic polymer material with the possibility of functionalization had to be chosen to prepare desirable filtration membranes. The immobilization of proper bioactive molecules (antibodies) secured the functionalization of nanofiber membranes. For the required application, specific antibodies were selected as a biorecognition element for detecting the model bacteria (*Escherichia coli* and *Staphylococcus aureus*). After preparing and characterizing the prepared bioreceptor, functionalized nanofiber membranes were tested in the laboratory.

### 2.1. Materials

Polymer polyacrylonitrile (PAN) was purchased from Sigma-Aldrich (USA) to fabricate electrospun nanofibers. This polymer was chosen due to its mechanical and chemical endurance and the possibility of surface functionalization. The functionalization of PAN nanofibers was performed by the immobilization of specific antibodies. For the interception of Gram-negative model bacteria, Rabbit polyclonal IgG anti-*Escherichia coli* antibodies (4329–4906) were purchased from Bio-Rad (USA). Anti-*Staphylococcus aureus* LTA antibodies (SAB4200883-100UL) from Sigma-Aldrich (USA) were immobilized to nanofibers to detect the Gram-positive model bacteria *Staphylococcus aureus*.

The University of Chemistry and Technology, Prague, provided Gram-negative model bacteria *Escherichia coli* reference strains (O26:B6, *E. coli* DBM 3125—collection CCM 3988). The Institute of Medical Biochemistry and Laboratory Diagnostics, First Faculty of Medicine, Charles University in Prague, provided Gram-positive bacteria *Staphylococcus aureus* (STAV) strains.

### 2.2. Nanofiber Fabrication, Modification, and Characterization

For the biosensor matrix, polyacrylonitrile nanofibers were fabricated using the electrospinning method. Electrospinning uses the charge polymer solution under a high-voltage electric field to prepare ultrafine fibers with diameters of hundreds of nanometers [34]. Electrospun nanofibers are characterized by extremely high surface-to-volume ratio, high porosity, low weight, and excellent mechanical and chemical properties. Nevertheless, all the properties can be customized by adequately selecting a polymer solution and setting the process parameters of the fabrication method [19,35,36].

Polyacrylonitrile (PAN) polymer is suitable for preparing fine nanofibers with excellent mechanical and chemical stability. Fibers fabricated from polyacrylonitrile are ideal for filtration and the creation of biosensor matrices (mats). These fibers are also suited for surface functionalization by immobilizing bioactive molecules [37,38].

**Figure 1:**
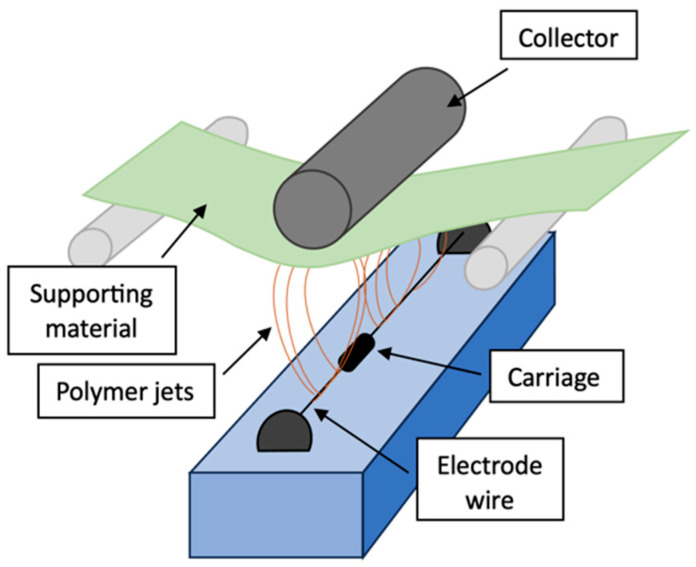
Setup of Nanospider device for fabrication of electrospun nanofibers [33].

To fabricate suitable nanofibers, the powder of polyacrylonitrile was mixed with N, N-dimethylformamide (DMF) and homogenized for 2 h at 35 °C. Electrospun PAN nanofibers were fabricated (roller electrospinning—Figure 1) using Nanospider NS 1WS500U (Elmarco, Liberec, Czech Republic). The process parameters are shown in Table 1.

After fabrication, samples of nanofibers were gilded and characterized through the scanning electron microscope Vega3 SB (Tescan, Brno, Czech Republic).

The created nanofibers were later modified and functionalized. PAN nanofibers’ surface modification (reduction) ensures the formation of functional groups suitable for bonding bioactive molecules [39]. Specific antibodies against bacteria *E. coli* and *S. aureus* were then covalently immobilized in the structure of PAN nanofibers. The concentration of bonded antibodies was determined by infrared spectroscopy IRAFfinity-1 (Shimadzu, Kyoto, Japan), and the absorbance of 1685 cm^−1^, characteristic of the peptide bond, was used. The calibration curve was determined using avidin and measuring the remaining protein in the solution after immobilization [40,41].

The functionalized nanofibers were prepared and preserved in a saline buffer with sodium azide. Samples preserved this way were stored in the fridge. Previous testing shows that preserved functionalized nanofiber membranes can be stored in the fridge for at least 2 months without changing the antibody activity.

### 2.3. Bacterial Cultivation

Both model organisms—*E. coli* and *S. aureus*—were cultured on a solid agar medium prepared from 2.5 g of yeast extract, 2.5 g of peptone, 1.125 g of NaCl, and 5 g of agar. Individual media components were mixed in 250 mL of distilled water, homogenized, heated, and sterilized before being poured into the Petri dishes.

From the reference strains, a single colony of bacteria was transferred to the agar medium using the streak plate method; passaged bacteria were cultured at 37 °C in the incubator (mini-incubator ICT 18, FALC Instruments, Treviglio BG, Italy). *E. coli* was incubated for 21 h and *S. aureus* for 24 h to achieve adequately grown bacterial colonies.

### 2.4. Testing of the Nanofiber Bioreceptor

**Figure 2:**
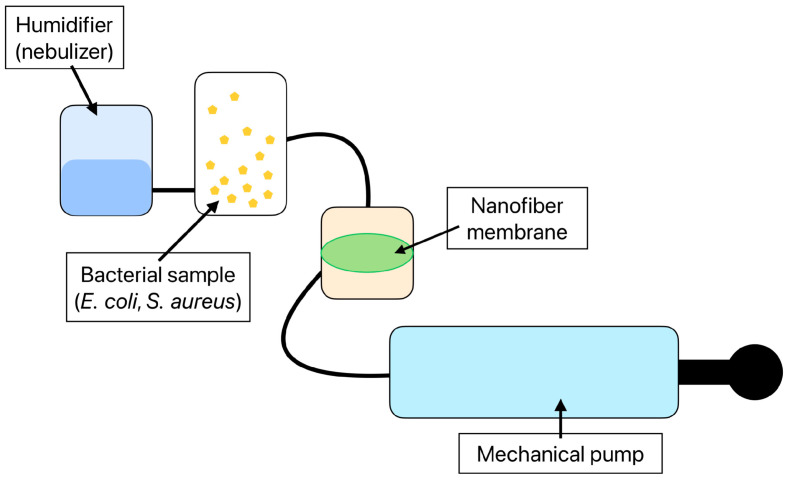
The layout of the air filtration system consisting of a mechanical pump, a 1.5 l sample container, a sealed container with a nanofiber membrane, and a humidifier sustaining the proper environment for the antibody immobilized to the nanofiber structure [42].

A unique pump system was designed to test the detection effectivity of the functionalized nanofibers. The created system consists of a mechanical pump enabling the filtration of the air sample through the nanofiber membrane in a sealed chamber. A sample container with a volume of 1.5 l is connected directly to the sealed chamber. The whole pump system is closed and provided with filters and thus does not allow bacteria to escape from the experimental setup. Moreover, this unique pump system was designed to maintain suitable conditions for the immobilized antibodies by continually humidifying filtered air. The detailed layout (Figure 2) and function of the air filtration system are presented in the original paper from 2024 [42].

Nanofiber membranes were tested as a bioreceptor for the interception of bacterial cells. Before use, membranes were washed in distilled water so the saline buffer and preservative residues would not affect the detection. After washing, the nanofiber membrane was evenly spread to the holder in the sealed chamber. The volume of contaminated air in the sample container was then filtered through the functionalized nanofiber membranes using the mechanical pump. After the filtration, membranes were cleansed for 10 s in 1× PBS buffer to wash out bacterial cells that did not bind to the antibodies.

The functionalized PAN nanofibers as bioreceptors were tested in different conditions, namely dry air filtration and air filtration with additional humidification of the nanofiber membranes.

Nanofiber membranes were transferred to the liquid growth medium and incubated at 37 °C for 21 h (*E. coli*) or 24 h (*S. aureus*). After the incubation, 1 mL of homogenized bacterial suspension was transferred to the spectrophotometric cuvette. The bacterial suspensions’ optical density (wavelength 600 nm) was measured through the spectrophotometer UV-3600 (Shimadzu, Kyoto, Japan) to evaluate the number of captured bacteria. The parameters of the used spectrophotometer are shown in Table 2.

### 2.5. Data Analysis and Evaluation of Bioreceptor Effectivity

The bioreceptor effectivity evaluation dataset consists of 144 measurements for *E. coli* and 90 measurements for *S. aureus*. For both model organisms, three types of samples were used: functionalized nanofibers FNn for humid air filtration, FNs for dry air filtration, and unmodified nanofibers NN for humid air filtration. Using a series of samples ensured the reproducibility and repeatability of the experiments. The individual series were compared with each other, and the comparison was evaluated.

For *E. coli*, 24 nanofiber membranes (8 for each type) were used. A series of 15 nanofiber membranes were tested through air filtration polluted by the model organism *S. aureus*. After the air filtration through the membranes and membrane incubation, bacterial suspensions were created, and the optical density (OD_600_) was measured (spectrophotometer UV-3600, Shimadzu, Kyoto, Japan).

The optical densities dataset consists of six measured values for each nanofiber sample, ranging from OD_600_ of 0.164 to 1.677 for *E. coli* and OD_600_ of 0.456 to 1.132 for *S. aureus*. From these values, the mean and the median were calculated and then compared for each type of nanofiber membrane (FNn, FNs, and NN). In addition, the statistical significance (*p* = 0.05) of the obtained data was determined through the *t*-test.

R software with an EZR plug-in was used to analyze the data and graphically represent the results [43].

## 3. Results

### 3.1. Preparation and Characterization of PAN Nanofibers

PAN nanofibers were prepared using the roller electrospinning method (needleless electrospinning) and functionalized by immobilizing the specific antibodies. Due to the high surface-to-volume ratio, even a small part of the functionalized nanofiber obtains many antibodies. The final concentration of antibodies was determined by IR spectroscopy to be 108 ± 12 µM/g.

**Figure 3:**
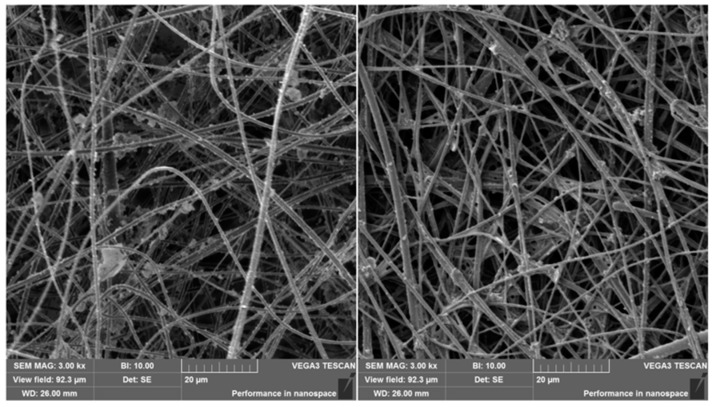
Surface-modified (**left**) and anti-*E. coli*-functionalized (**right**) PAN nanofibers.

The structure of PAN nanofibers was characterized through SEM. Predominantly regular fibers with a mean diameter between 500 and 900 nm were observed (Figure 3).

From the prepared nanofibers, circle membranes with a diameter of 1.5 cm were cut. Due to the use of a 3D-printed stand for the nanofiber membranes, the real functional diameter was limited to 1 cm (the part through which the air was filtered).

The nanofiber membranes were stored in a saline buffer, so the antibody was preserved. For longer preservation, sodium azide was added to the saline buffer. Before their use as filters, nanofiber membranes were washed from chemical residues and preservatives with distilled water.

### 3.2. The Detection of Bacteria and Evaluation of Bioreceptor Effectiveness

The effectiveness of the bacterial interception by nanofiber membrane was evaluated through the optical density of created bacterial suspensions. The obtained results are divided according to the detected model organisms in the following subsections:

#### 3.2.1. Detection of *Escherichia coli*

**Figure 4:**
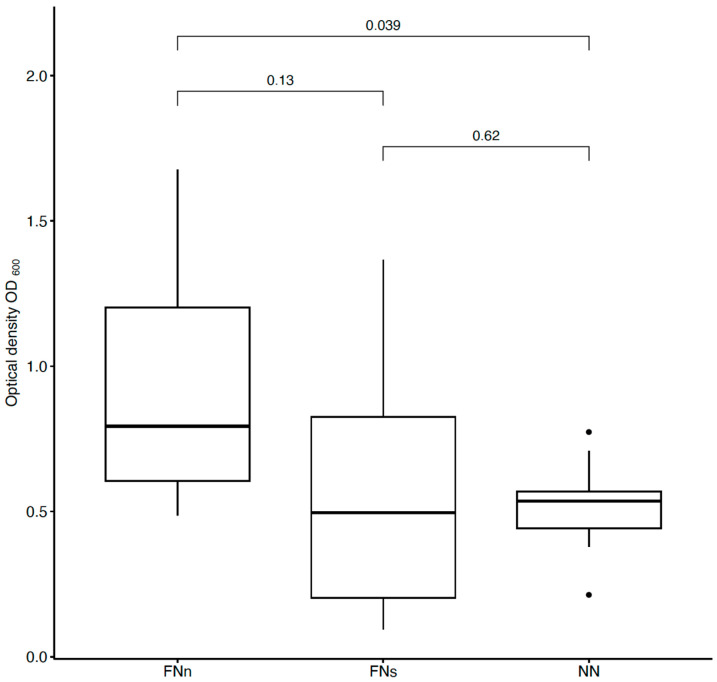
Comparison of bacterial suspensions’ optical densities OD_600_ created from *E. coli* cells captured into the nanofiber structure during humid air and dry air filtration. In the figure, FNn (anti-*E. coli* PAN nanofibers) and NN (unmodified PAN nanofibers) show the data obtained during humid air filtration and FNs (anti-*E. coli* PAN nanofibers) during dry air filtration. The numbers above the boxplots show the *p*-values.

To detect *E. coli* bacteria from sufficiently humid air (an average of 60%), unmodified and anti-*E. coli* PAN nanofiber targets were used and compared (Figure 4). In addition, filtration under different conditions was tested. To determine the extent of the proper environment, anti-*E. coli* PAN nanofibers were used to detect bacteria during humid air and dry air filtration, and the bacterial interception was compared (Figure 4). The measurements were divided into eight series always consisting of the three samples (FNn, FNs, and NN).

**Figure 5:**
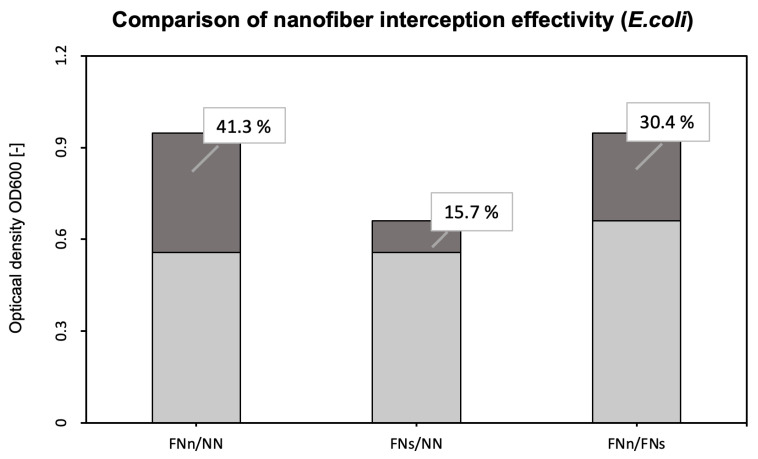
Comparison of the interception effectivity for functionalized and unmodified nanofibers and two types of filtrations. The dark part and percentages show the increase in the effectivity of functionalized nanofibers (FNn and FNs) compared to unmodified nanofibers NN (FNn/NN for humid air filtration and FNs/NN for dry air filtration). The third column shows the increase in interception effectivity of functionalized nanofibers during humid air filtration (FNn) compared to dry air filtration (FNs).

For better clarity, Figure 5 compares the filter effectiveness between unmodified and functionalized (FNn/NN and FNs/NN) nanofiber membranes and the two used filtration methods under different conditions (FNn/FNs).

#### 3.2.2. Detection of *Staphylococcus aureus*

**Figure 6:**
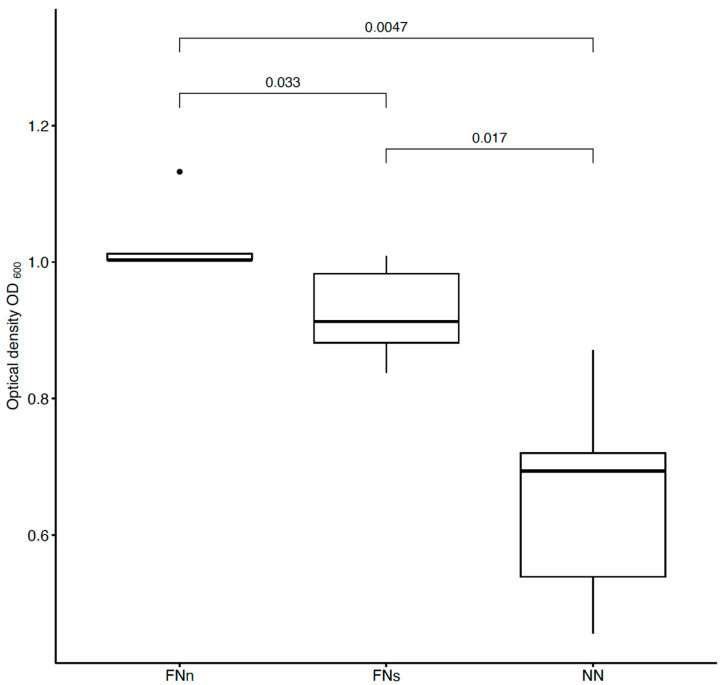
Comparison of bacterial suspensions’ optical densities OD_600_ created from *S. aureus* cells captured into the nanofiber structure during humid air and dry air filtration. In the figure, FNn (anti-*S. aureus* PAN nanofibers) and NN (unmodified PAN nanofibers) show the data obtained during humid air filtration, and FNs (anti-*S. aureus* PAN nanofibers) during dry air filtration. The numbers above the boxplots show the corresponding *p*-values.

As explained previously for bacteria *E. coli*, two experiments were performed for Gram-positive bacteria *Staphylococcus aureus*. Functionalized anti-*S. aureus* PAN nanofibers (FNn) were compared to the unmodified ones (NN). In addition, a comparison of the bacterial interception of the functionalized nanofibers under different conditions was performed. The estimated optical densities of both experiments are shown in Figure 6. Five series consisting of the three nanofiber samples (FNn, FNs, and NN) were evaluated.

**Figure 7:**
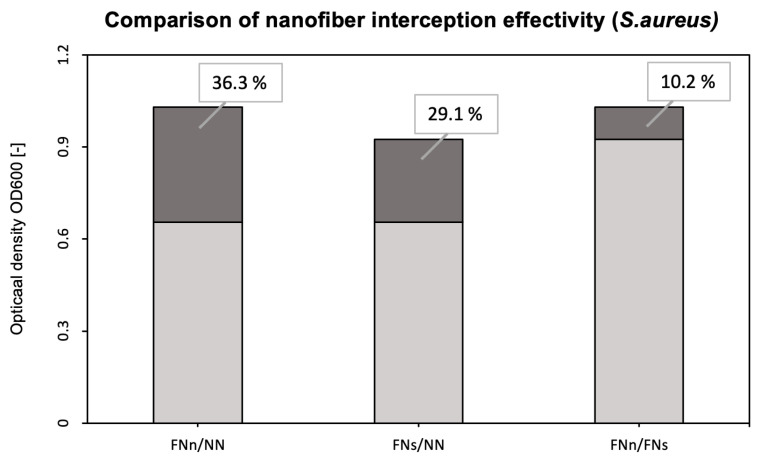
Comparison of the interception effectivity for functionalized and unmodified nanofibers and two types of filtrations. FNn/NN shows the difference in the interception effectivity of the functionalized and unmodified nanofibers during humid air filtration. FNs/NN shows the same difference but during dry air filtration. The FNn/FNs column then shows the increase in interception effectivity of functionalized nanofibers during humid air filtration (FNn) compared to dry air filtration (FNs).

A more detailed comparison of the interception effectivity is shown in Figure 7.

## 4. Discussion

This work presents the creation and the bacterial interception effectivity evaluation of a novel immunoreceptor based on antibody-functionalized PAN nanofibers. To detect airborne bacteria (*E. coli* and *S. aureus*) directly from the air, electrospun nanofibers with great mechanical and chemical durability were used as filtration membranes. PAN nanofibers were selected due to their exceptional filtration ability and the possibility of surface functionalization. Although electrospun PAN nanofiber membranes are capable of bacterial interception itself and with great effectivity (up to 99%) [44], antibody-functionalized nanofibers capture bacterial cells with specific biochemical bonds (antigen-antibody reaction). In the case of nanofiber bioreceptors, the mechanical interception of bacterial cells is undesirable due to the rapid clogging of the filtration membranes. In comparison with a previous study dealing with the filtration effectivity of PAN nanofibers [44], the area density of functionalized membranes for bacterial detection was reduced to 2.5 g/m^2^, so the mechanical interception was suppressed.

As mentioned earlier, PAN nanofibers were functionalized by immobilizing specific antibodies against *E. coli* and *S. aureus*. Bioactive molecules, such as antibodies, used as a biosensing layer of biosensors are dependent on stable and specific conditions (temperature, pH, humidity, and electrostatic repulsion). When detecting antigens directly from the air, humidity is the most challenging condition to maintain. Without additional moisturization, immobilized antibodies lose their bioactivity [45,46]. For this reason, bacterial detection, whether using conservative methods (ELISA, PCR, etc.) or (bio)sensors, is performed in liquid samples (water, body fluids, food, etc.) [26,29,30,47,48]. Airborne samples, thus, must undergo post-collection processing [31,49,50,51]. However, with the use of a previously designed air filtration system [42], the presented nanofiber bioreceptor was used and tested for the detection of bacterial cells directly from the air. This system humidifies nanofiber membranes during air filtration and protects immobilized antibodies from desiccations and, thus, inactivation (Figure 4 and Figure 6) [42].

To evaluate the bioreceptor effectiveness, bacterial interception through unmodified and functionalized nanofibers was compared. The increase in the optical density of bacterial suspensions (around 41 % for *E. coli* and 36 % for *S. aureus*, as seen in Figure 5 and Figure 7) belonging to the functionalized nanofiber membranes testing shows the effectivity of immobilized antibodies (the specific binding reaction of the bioreceptor). For both model organisms, the increase in interception effectivity due to the antibodies’ activity was found to be statistically significant at the significance level of *p* < 0.05. Thus, in comparison with other mentioned nanofiber biosensors for bacterial detection [26,27,28], the designed nanofiber bioreceptor combines both biosensing and filtration abilities. In further research, a combination of such a bioreceptor with a proper transducer could be a pioneering alternative for fast, sensitive, and continual environment monitoring presented in recent years [52,53,54,55].

As in other studies [56,57,58], PAN nanofibers have been proven to be membranes with extraordinary air filtration abilities. After enrichment by metal particles (TiO_2_, ZnO, Ag, etc.) [57] or bioactive molecules (enzymes and antibodies), PAN membranes show additional abilities, such as antibacterial [57] or biosensing activity, in relation to bacteria. Presented antibody-functionalized PAN nanofibers, thus, show great potential as a novel sensitive bioreceptor for detecting Gram-negative and Gram-positive bacteria such as *E. coli* and *S. aureus*.

## 5. Conclusions

Herein, we presented the preparation and use of the novel antibody-functionalized PAN nanofibers as bioreceptors for bacterial detection from the air. To detect model bacterial organisms *E. coli* and *S. aureus*, PAN nanofiber membranes were fabricated through the needleless electrospinning process and later functionalized by immobilizing corresponding antibodies. The specific structure of electrospun nanofibers enables the use of the membranes for air filtration. In addition, antibody functionalization significantly increases the bacterial interception effectivity of the membrane (on average about 40%) and facilitates the formation of special biochemical bonds with detected antigens (bacteria). In combination with the system for air filtration presented in previous work, the designed antibody-functionalized PAN nanofiber bioreceptor enables reliable, specific, and sensitive detection of Gram-negative and Gram-positive bacteria directly from the air and without inactivation and disintegration of the immobilized bioactive layer. Our finding opens the door for the development of a novel solution for continual environment monitoring. In addition, further studies will focus on combining the presented bioreceptor with a suitable electrode and the development of an ultrasensitive biosensor for bacterial detection.

## Figures and Tables

**Table 1:** Set process parameters of the electrospinning (with the deviation given by the Nanospider NS 1WS500U device) [33].

Fabrication Parameters	Values
Solution	PAN + DMF
Solution concentration [%]	15
Diameter of the wire electrode [mm]	0.2
Distance between electrodes [cm]	25
Temperature [°C]	20
Relative humidity [%]	20
Voltage [kV]	50–90

**Table 2:** Spectrophotometer hardware parameters [33].

Hardware Parameters	Values
Wavelength range [nm]	185–3300
Wavelength accuracy for UV and VIS [nm]	±0.2
Wavelength accuracy for IR [nm]	±0.8
Photometric range [Abs]	−6–6
Photometric accuracy [Abs] for 1 Abs	±0.003
Photometric accuracy [Abs] for 0.5 Abs	±0.002

## Data Availability

The data presented in this study are available upon request.
